# Caudo-rostral brain spreading of α-synuclein through vagal connections

**DOI:** 10.1002/emmm.201302475

**Published:** 2013-05-23

**Authors:** Ayse Ulusoy, Raffaella Rusconi, Blanca I Pérez-Revuelta, Ruth E Musgrove, Michael Helwig, Bettina Winzen-Reichert, Donato A Di Monte

**Affiliations:** German Center for Neurodegenerative Diseases (DZNE)Bonn, Germany

**Keywords:** Adeno-associated virus, Parkinson's disease, protein transport, rat, vagus nerve

## Abstract

α-Synuclein accumulation and pathology in Parkinson's disease typically display a caudo-rostral pattern of progression, involving neuronal nuclei in the medulla oblongata at the earliest stages. In this study, selective expression and accumulation of human α-synuclein within medullary neurons was achieved *via* retrograde transport of adeno-associated viral vectors unilaterally injected into the vagus nerve in the rat neck. The exogenous protein progressively spread toward more rostral brain regions where it could be detected within axonal projections. Propagation to the pons, midbrain and forebrain followed a stereotypical pattern of topographical distribution. It affected areas such as the coeruleus–subcoeruleus complex, dorsal raphae, hypothalamus and amygdala ipsilateral and, to a lesser extent, contralateral to the injection side. Spreading was accompanied by evidence of neuritic pathology in the form of axonal varicosities intensely immunoreactive for human α-synuclein and containing Thioflavin-S-positive fibrils. Thus, overexpression of human α-synuclein in the lower brainstem is sufficient to induce its long-distance caudo-rostral propagation, recapitulating features of Parkinson's disease and mechanisms of disease progression.

## INTRODUCTION

Brain accumulation, aggregation and spreading of α-synuclein (α-syn) are hallmarks of Parkinson's (PD) and other neurodegenerative diseases (Spillantini et al, 1997). Although the exact mechanisms triggering this α-syn pathology are yet to be fully elucidated, both clinical and experimental evidence is consistent with a key role of increased α-syn expression as a causative or predisposing factor in disease pathogenesis. Indeed, a life-long elevation of α-syn expression due to multiplication mutations of its gene causes familial parkinsonism; other conditions, such as aging and neuronal injury, associated with more transient α-syn increases could also promote α-synuclein pathology and enhance disease risk (Ross et al, 2008; Simon-Sanchez et al, 2009; Ulusoy and Di Monte, 2013). A significant feature of α-syn pathology in PD is its pattern of ascending progression. Typically, early targets of α-syn accumulation are neurons in the medulla oblongata (MO), particularly in the dorsal motor nucleus of the vagus nerve (DMnX) and the reticular formation (Braak et al, 2003a; Braak et al, 2003b). During disease progression, α-syn pathology spreads upwardly toward the pons, mesencephalon and higher brain regions, following a stereotypical pattern that may reflect neuron-to-neuron transmission (Desplats et al, 2009; Hansen et al, 2011; Freundt et al, 2012) and propagation *via* interconnected brain pathways (Braak et al, 2003a; Luk et al, 2012a; Luk et al, 2012b). The reason(s) that underlie the involvement of MO neurons in the early stages of α-syn accumulation are not fully understood. It has been hypothesized, however, that pathogenic forms of the protein may initially reach the MO while being carried from peripheral sites (e.g., enteric plexi) to the CNS through the vagus nerve (Braak et al, 2003b; Pan-Montojo et al, 2012).

Caudo-rostral spreading of α-syn from the lower brainstem remains to be demonstrated experimentally, and no animal model is currently available that directly mimics this important PD feature. Furthermore, it is unclear whether increased expression of α-syn would itself be capable of causing long-distance upward transmission of the protein and its pathology. To address these unanswered questions, a new model of targeted elevation of neuronal α-syn in the rat MO was first developed. Using this model, we were then able to show progressive caudo-rostral α-syn propagation and pathological protein accumulation within dystrophic axons.

## RESULTS AND DISCUSSION

An experimental strategy was designed by which human α-syn (hα-syn) could be specifically overexpressed in the rat MO and then “traced forward” to assess its potential spreading. Recombinant adeno-associated viral vectors (AAV) expressing either wild-type hα-syn or green fluorescent protein (GFP) were injected into the left vagus nerve in the rat neck with the intent of transducing MO neurons through retrograde viral transport (Towne et al, 2010).

**Figure 1:**
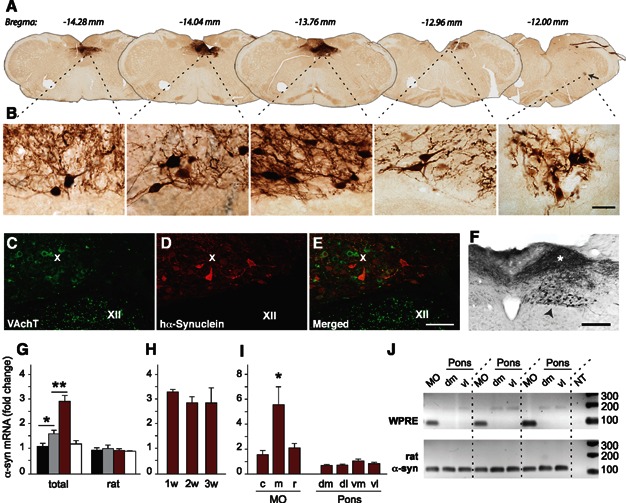
Vagal injections of hα-syn-carrying AAV induce region-specific transduction **A,B.**Representative MO sections from a high expressor rat killed at 2 weeks post viral injection were stained for hα-syn. Caudo-rostral sections at corresponding Bregma levels were visualized at lower (A) and higher (B) magnification. The nucleus ambiguus (arrow) is visible in the section at Bregma −12.00 mm. Higher-magnification images show neuronal bodies and neurites in the DMnX and nucleus ambiguus. Scale bar, 50 μm.**C-E.**A representative section of the MO from a high expressor rat was double-stained with anti-vesicular acetylcholine transporter (VAchT) and anti-hα-syn antibodies. Confocal images show an area comprising neurons in the DMnX (X) and hypoglossal nucleus (XII). While VAchT immunoreactivity labeled neurons in both nuclei (green), hα-syn staining was only present in the DMnX (red) where it co-localized with the cholinergic marker (merged image). These observations are consistent with targeted transduction *via* vagus nerve projections. Scale bar, 200 μm.**F.**A representative section of the MO from a high expressor rat was immunostained with an anti-hα-syn antibody. Arrowhead indicates cell bodies in the left (ipsilateral to viral injection) DMnX. The asterisk highlights neuronal projections immunoreactive for hα-syn at the level of the nucleus of the tractus solitarius. Some of these projections cross the mid-line and innervate the contralateral MO. Scale bar, 100 μm.**G.**Analyses were made at 2 weeks post viral injection. For each rat, DMnX-containing samples from the caudal, middle and rostral MO were combined. qRT-PCR analyses measured total (rat + human) and rat-only α-syn mRNA levels. Data are shown from (i) the MO of control rats (black bars, n = 6), (ii) the left (AAV-injected side) MO of low expressor animals (gray bars, n = 4), (iii) the left (AAV-injected side) MO of high expressor rats (red bars, n = 10), and (iv) the right MO (contralateral to the AAV injection) of high expressors (empty bars, n = 4). ANOVA and *post hoc t* test with Bonferroni correction, F_3,20_ = 17.80. Mean ± SEM. *P < 0.05 and **P < 0.01.**H.**Total (rat + human) α-syn mRNA levels were compared by qRT-PCR at 1, 2 and 3 weeks post injection in DMnX-containing samples from the left (AAV-injected side) MO of high expressor rats (n ≥ 3/group). Tissue was collected as described above in G. Mean ± SEM.**I.**Total α-syn mRNA levels were measured by qRT-PCR in DMnX-containing samples from the left (AAV-injected side) caudal (c), middle (m) or rostral (r) MO of high expressor rats. ANOVA and Tukey *post hoc* test, F_2,14_ = 6.409. Analyses were also made in samples from the dorso-medial (dm), dorso-lateral (dl), ventro-medial (vm) and ventro-lateral (vl) pons. Mean ± SEM. *P < 0.05.**J.**WPRE and rat-only α-syn mRNA was amplified by RT-PCR in DMnX-containing samples from the left (AAV-injected side) MO of high expressor rats (n = 3). Analyses were also made in samples from the left dorso-medial (dm) and ventro-lateral (vl) pons. Specific bands were detected at 85 (WPRE) and 117 (rat α-syn) bp. NT labeling indicates non-template controls.

Robust staining of MO tissue with a specific hα-syn (Fig 1) or GFP (Supporting Information Fig 1) antibody at 2 weeks post-injection indicated successful transduction via cranial nerve X. The pattern of immunoreactivity was consistent with targeted expression since it followed the predicted anatomical distribution of efferent and afferent fibers forming the vagus nerve. Efferent projections originate in the DMnX and the nucleus ambiguus. Consequently, retrograde viral transport through these fibers resulted in robust transgene (hα-syn or GFP) expression within somata and neurites of these nuclei (Fig 1, Supporting Information Fig S1). Cholinergic DMnX cells could be double-stained with antibodies against the vesicular acetylcholine transporter and hα-syn, further confirming targeted transduction (Fig 1). Transgene expression within neuronal cell bodies was only observed in the DMnX and nucleus ambiguus ipsilateral to AAV injection. This is consistent with the results of earlier anatomical studies showing unilateral labeling of DMnX perikarya after injections of tracers into the rat vagus nerve (Leslie et al, 1982).

Afferent vagal fibers projecting to the dorsal MO originate from sensory neurons in the inferior vagal ganglion from where they reach the nucleus of the tractus solitarius. Viral transduction of these cells was indicated by strong hα-syn staining of axonal bundles occupying an area dorsal to the DMnX (Fig 1). Labeled fibers were mostly observed ipsilateral to the AAV injection. However, in agreement with previous reports demonstrating bilateral terminal fields of vagal sensory afferents (Leslie et al, 1982; Kalia & Sullivan, 1982; Odekunle & Bower, 1985), some of these axons crossed the midline and innervated the contralateral MO (Fig 1). The pattern of distribution of efferent and afferent vagal fibers in the MO was virtually undistinguishable between animals injected with hα-syn- or GFP-carrying AAV vectors (Fig 1, Supporting Information Fig S1).

The majority of rats (>60%) displayed evidence of strong transduction, with a significant number of stained cell bodies and high density of immunoreactive neurites (Fig 1). qRT-PCR performed on DMnX-containing MO tissue from these high expressor animals revealed a 2.9-fold increase in total (rat plus human) α-syn mRNA; rat-only α-syn expression was unchanged (Fig 1). The extent of neuronal immunoreactivity was less pronounced in a second group of animals (Supporting Information Fig S2), suggesting a less effective transduction with viral DNA. Total α-syn mRNA was 1.6 fold higher in the MO of these rats as compared to control tissue (Fig 1). MO samples were also collected at 1 week and 3 weeks post-injection; qRT-PCR analyses demonstrated that maximal transduction was already reached at 1 week and remained unchanged at 2 and 3 weeks (Fig 1).

Three sets of experiments were designed to further define the distribution of AAV transduction in high expressor rats. In the first set, total α-syn mRNA was quantified by qRT-PCR in DMnX-containing MO tissue from the side of the brain contralateral to viral injection; data showed levels not significantly different than control values (Fig 1). Then, qRT-PCR analysis was performed on samples from different caudo-rostral portions of the MO and different quadrants of the pons. Total α-syn mRNA was enhanced in the MO, particularly its middle portion (Fig 1). In contrast, no changes occurred in the pons, indicating absence of virus-encoded hα-syn mRNA (Fig 1). The genome of our AAV vector also encoded for the woodchuck hepatitis virus post-transcriptional regulatory element (WPRE). Therefore, in the third set of experiments, MO and pontine tissue was analyzed for expression of this enhancer element using WPRE-hybridizing primers. RT-PCR results confirmed the occurrence of AAV transduction in the MO but found no evidence of WPRE mRNA in the dorso-medial and ventro-lateral (Fig 1) as well as dorso-lateral and ventro-medial (not shown) pons.

**Figure 2:**
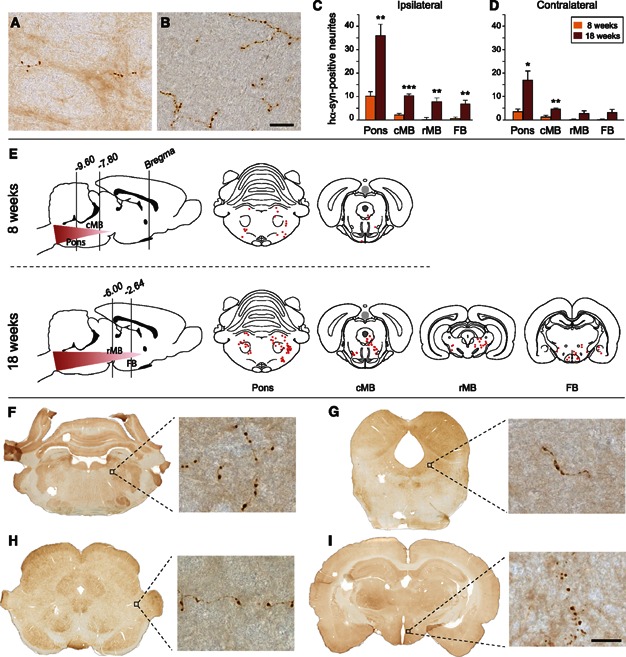
Hα-syn progressively spreads from the MO to more rostral brain regions **A,B.**Representative images of hα-syn-immunoreactive axons in tissue sections from the pons of high expressor rats sacrificed 8 (A) or 18 (B) weeks after vagal injection. Scale bar, 25 μm.**C,D.**The number of neuritic projections immunostained with an anti-hα-syn antibody was counted in the pons, caudal midbrain (cMB), rostral midbrain (rMB) and forebrain (FB) at 8 and 18 weeks post injection. Counts are from the side of the brain ipsilateral (C) and contralateral (D) to viral injection. Mean (n = 5/group) ± SEM. *P < 0.05, **P < 0.01, ***P < 0.001 by two-tailed *t* test.**E.**Topographical plot of the distribution and spreading of hα-syn-labeled neurites. Neuronal fibers from representative brain sections are shown as red dots. Bregma values indicate the caudo-rostral level.**F–I.**Representative tissue sections from the pons (F), caudal midbrain (G), rostral midbrain (H) and forebrain (I) of high expressor rats sacrificed at 18 weeks post vagal injection. Higher magnification images from the coeruleus-subcoeruleus complex (F), periacqueductal gray (G), peripeduncular nucleus (H) and hypothalamus (I) show axonal projections stained with an anti-hα-syn antibody. Scale bar (higher magnification panels), 25 μm.

The rat vagus nerve is comprised of axons that originate from or terminate in the MO and upper cervical spinal cord (Kalia & Sullivan, 1982). Thus, under our experimental conditions, presence of transduced protein in brain regions rostral to the MO would be consistent with interneuronal upward spreading. To test this possibility, we analyzed post-mortem histological sections throughout the brain of rats killed at 4, 8 or 18 weeks after vagal injection. Hα-syn or GFP immunoreactivity remained confined to the MO at the earliest time point. After longer post-injection intervals, however, evidence of ascending protein propagation characterized the brain of rats with high hα-syn expression. The exogenous protein was detected within discrete axonal projections that became immunoreactive for hα-syn. Labeled fibers were already seen in the pons and caudal midbrain at 8 weeks (Fig 2). At 18 weeks, they were significantly more numerous and also occupied the rostral mesencephalon and prosencephalic areas (Fig 2).

Progressive hα-syn spreading affected not only the left side of the brain (ipsilateral to viral injection) but also the contralateral pons, midbrain and forebrain; the count of immunoreactive axons was approximately 35% in the right as compared to the left hemisphere (Fig 2). The most likely explanation for this intriguing finding relates to the bilateral distribution of axons that project from higher brain regions to nuclei in the MO (van der Kooy et al, 1984). Through these projections, the hα-syn released from neurons in the left MO could be taken up and transported to ipsilateral as well as contralateral sites.

Spreading followed a stereotypical pattern and sequence of topographical distribution (Fig 2). Predilection loci included the pontine coeruleus–subcoeruleus complex (Fig 2) where hα-syn-containing neurites could be double-labeled for tyrosine hydroxylase (Supporting Information Fig S3). In the midbrain, hα-syn-labeled fibers were observed in the dorsal raphae, periacqueductal gray and in the area of the peripeduncular nucleus dorsolateral to the substantia nigra (SN) pars reticulata (Fig 2). Other typical sites were the hypothalamus in the diencephalon (Fig 2) and the amygdala in the medial temporal lobe. All of these areas share a relevant feature, i.e. direct projections into the MO (Ter Horst et al, 1991; van der Kooy et al, 1984), supporting a mechanism of hα-syn transmission *via* anatomically interconnected pathways.

Results in high expressor rats, as described so far, contrasted with findings in animals with more moderate hα-syn expression and in rats injected with GFP-carrying AAV. The former displayed a few immunoreactive fibers only in the pons and only at the 18-week time point (Supporting Information Fig S4). The latter showed no significant GFP propagation.

**Figure 3:**
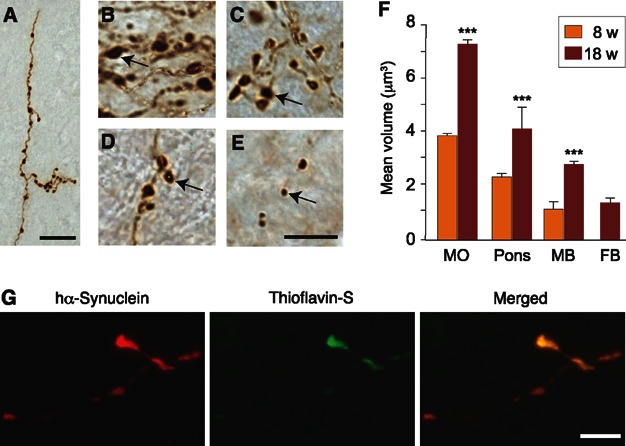
Spreading of hα-syn is associated with neuritic pathology **A.**Brain sections from a high expressor rat killed 18 weeks after vagal injection were stained with an anti-hα-syn antibody. The representative image shows a pontine axon with intensely stained swellings. Scale bar, 20 μm.**B–E.**Hα-syn-immunoreactive neuritic varicosities in different brain regions at 18 weeks post injection. Arrows indicate swellings of different sizes in the MO (B, 7.3 μm^3^), pons (C, 4.1 μm^3^), midbrain (D, 2.8 μm^3^) and forebrain (E, 1.4 μm^3^). Scale bar, 10 μm.**F.**The volume of hα-syn-immunoreactive neuritic swellings was measured in the MO (n = 1568 and 2406 swellings at 8 and 18 weeks, respectively), pons (n = 357 and 461 at 8 and 18 weeks), midbrain (n = 8 and 296 at 8 and 18 weeks) and forebrain (n = 34 at 18 weeks). Mean ± SEM. ***P < 0.001 by Wilcoxon Rank Sums test.**G.**Confocal images of a pontine axon stained with an anti-hα-syn antibody (red) and Thioflavin-S (green). Merged images show co-localization. Scale bar, 5 μm.

Accumulation of hα-syn in the MO as well as extra-medullary sites was accompanied by morphological evidence of neuronal abnormalities. In particular, hα-syn-containing axons often appeared as sinuous threads with irregularly spaced, densely labeled varicosities (Fig 3). The volume of these swellings augmented over time and was more pronounced in caudal *vs*. rostral brain regions, consistent with increasing hα-syn burden and progressive neuritic pathology (Fig 3). Staining with Thioflavin-S and co-localization of Thioflavin-S with hα-syn immunoreactivity was used to assess neuronal content of hα-syn amyloid fibrils. In the pons, a small percentage of hα-syn-labeled neurites were stained with Thioflavin-S at 8 weeks. Co-localization characterized a higher proportion of pontine fibers at 18 weeks (Fig 3), when rare Thioflavin-S-positive neurites were also seen in midbrain and forebrain sections. In all instances, staining with Thioflavin-S detected hα-syn fibrils primarily within neuritic swellings.

No evidence of hα-syn spreading and no overt sign of neuronal damage were found in the SN pars compacta (SNc). Stereological counting of dopaminergic neurons in rats killed 18 weeks after vagal injection confirmed that the cell number was unchanged in the left SNc of rats injected with hα-syn-carrying AAV (10,500 ± 452.6) as compared to values (i) in the right SNc (10,633 ± 427.3) from the same animals, and (ii) in the left and right SNc of control rats injected with GFP-carrying AAV (10,343 ± 449.5, left; 10.417 ± 435.8, right) (ANOVA, F_3,12_ = 0.07968, *P* = 0.9698).

The animal model described in this study provides experimental support in favor of the hypothesis that accumulation of α-syn in the MO can trigger further build-up and transmission of the protein toward more rostral CNS regions. Significant features of hα-syn spreading in rats include (i) the targeting of preferred anatomical sites, (ii) a consistent sequence of histological progression, and (iii) bilateral but asymmetric brain burden. These observations are noteworthy since they resemble important aspects of α-syn pathology in PD.

Other findings of this study are apparently less consistent with the human disease. For example, while propagation of hα-syn proceeded through axonal projections, no evidence of accumulation of the exogenous protein was found within neuronal cell bodies. Furthermore, mapping of hα-syn throughout the rat brain revealed its absence in the SNc. These two results, which are at odds with the occurrence of α-syn-containing inclusions within neuronal somata and the vulnerability of nigral dopaminergic cells to α-syn pathology in PD, may underscore limitations of the animal model. It is also quite possible, however, that features described above recapitulate early events in the spreading of α-syn leading to its pathological accumulation. Indeed, previously published experimental work supports the notion that α-syn pathology affects first axonal projections and then neuronal cell bodies, mimicking a retrograde progression of degenerative changes that has also been proposed for PD (Decressac et al, 2012, Cheng et al, 2010). The absence of hα-syn in the SNc during its initial spreading from the MO could reflect the lack of direct anatomical connections between these two brain regions. Follow-up studies are warranted to determine if spreading of the exogenous protein would ultimately reach the SNc at later (>18 weeks post AAV injection) time points.

Results of this study bear significant implications concerning mechanisms and consequences of interneuronal α-syn transmission. Previous work *in vitro* and *in vivo* has shown cell-to-cell passage of soluble α-syn (Desplats et al, 2009; Hansen et al, 2011). Here, we document long-distance α-syn spreading that is triggered by overexpression of the protein in a concentration-, time- and connectivity-dependent fashion. As importantly, build-up and propagation of α-syn was accompanied by morphological evidence of neuritic injury and increasing formation of fibrillar deposits. Luk and colleagues (2012a,b) have recently reported that, once misfolded fibrillar forms of α-syn are inoculated directly into the rodent brain, they induce a prion-like spreading of α-syn pathology associated with frank neurodegeneration. This important observation together with our present findings raises the possibility of the following scenario. Enhanced concentration, interneuronal transmission and aggregation of α-syn could set up a self-perpetuating loop that, fueled by the generation of toxic protein fibrils, may ultimately underlie the progression of α-syn pathology and neurodegenerative cascade in PD.

## MATERIALS AND METHODS

### Vectors

Recombinant adeno-associated virus (serotype 2 genome and serotype 6 capsid, AAV) was used for transgene expression of human α-synuclein (hα-syn) or enhanced green fluorescent protein (GFP) under the control of the human Synapsin1 promoter. Gene expression was enhanced using a woodchuck hepatitis virus post-transcriptional regulatory element (WPRE) and a polyA signal (Ulusoy et al, 2012). AAV vector production, purification, concentration and titration were performed by Vector Biolabs (Philadelphia, PA). Vector preparations were stored in PBS with 5% glycerol at −80°C until use. Titration of the concentrated vectors was performed using quantitative PCR and primers against WPRE. Injected titers for AAV-hα-syn and AAV-GFP were 1.0 × 10^13^ and 1.8 × 10^13^ genome copies/ml, respectively.

### Surgical procedure

Young adult female Sprague Dawley rats weighing 200–250 g were obtained from Charles River (Kisslegg, Germany). Animals were housed under a 12 h light/12-h dark cycle with free access to food and water. Experimental protocols were approved by the ethical committee of the State Agency for Nature, Environment and Consumer Protection in North Rhine Westphalia. Surgical procedures were performed on animals anesthetized with 2% isoflurane mixed with O_2_ and N_2_O. A 2 cm-incision was made at the midline of the rat neck, and the left vagus nerve was isolated from the surrounding tissue. A paraffin plate was placed underneath the nerve to provide support during injection and to prevent viral contamination of the surrounding tissue. Vector solution (2 µl) was injected at the rate of 0.5 µl/min using a glass capillary with a tip diameter of 60 µm fitted to a 5 µl Hamilton syringe. The capillary was kept in place for 3–4 minutes after injection.

### Tissue preparation

Animals were killed under pentobarbital anesthesia and perfused through the ascending aorta with saline, followed by ice-cold 4% (w/v) paraformaldehyde. Brains were removed, immersion-fixed in 4% paraformaldehyde (for 24 h) and cryopreserved in 25% (w/v) sucrose solution. Forty-µm sections were cut on the coronal plane using a freezing microtome. Sections throughout the brain were stored at −20°C in phosphate buffer (pH 7.4) containing 30% glycerol and 30% ethylene glycol.

### Histology

All immunohistochemical stainings were performed on free-floating sections. Brain sections were rinsed with Tris-buffered saline (TBS, pH 7.6). For brightfield microscopy, quenching of the endogenous peroxidase activity was achieved by incubation in a mixture of 3% H_2_O_2_ and 10% methanol in TBS. Non-specific binding sites were blocked by incubation in TBS with 0.25% Triton-X-100 (TBS-T) containing 5% normal serum. Samples were then incubated overnight at room temperature in primary antibody solution containing 1% normal serum in TBS-T. The following primary antibodies were used: mouse anti-hα-syn clone syn211 (36-008, Millipore; 1:10,000 dilution for color reaction or 1:3,000 for fluorescent detection), chicken anti-GFP (ab13970, abcam; 1:10,000), rabbit anti-tyrosine hydroxylase (for double-staining in the pons, AB152, Millipore; 1:1,000), mouse anti-tyrosine hydroxylase (for stereological cell counting, MAB318, Millipore; 1:2,000) and guinea pig anti-vesicular acetylcholine transporter (AB1588, Millipore; 1:500). Sections were rinsed, incubated (for 1 h at room temperature) in appropriate biotinylated secondary antibody solution (Vector Laboratories; 1:200) and then treated with avidin-biotin-peroxidase complex (ABC Elite kit, Vector Laboratories). Color reaction was developed using 3,3′-diaminobenzidine kit (Vector Laboratories). Sections were mounted on coated slides and coverslipped with Depex (Sigma).

For double-labeling immunofluorescence, primary antibodies were used as described above. Hα-syn was detected using biotinylated horse-anti-mouse (BA2001, Vector Laboratories) and a streptavidin-conjugated Dylight fluorophore (Dylight 488 or Dylight 594, Vector Laboratories; 1:400). Tyrosine hydroxylase and vesicular acetylcholine transporter were detected directly with Dylight 594. Thioflavin-S treatment was performed on hα-syn-stained sections mounted on glass slides; it consisted of 8-min incubations in 0,05% Thioflavin-S (dissolved in water) followed by sequential differentiation in 80-95-95% ethanol (3 minutes each). Fluorescent-labeled slides were all coated with PVA-DABCO (Sigma). Observations were made under a Zeiss LSM 710 NLO confocal microscope using 488 and 561 nm lasers with sequential acquisition.

**The paper explained**

PROBLEM:

α-Synuclein accumulation is a cardinal feature of Parkinson's disease (PD), so much so that a pathological staging of disease severity, first proposed by Braak and colleagues, is based on the spreading of α-synuclein lesions throughout the brain. Interestingly, initial sites of protein accumulation are nuclei in the lower brainstem; from there, α-synuclein pathology propagates upwardly in a progressive fashion. No animal model of α-synuclein caudo-rostral spreading is currently available, limiting our ability to study and elucidate mechanisms involved in the accumulation and propagation of α-synuclein and ultimately in disease development.

RESULTS:

We were able to reproduce PD-like α-synuclein spreading from the lower brainstem toward more rostral regions in the rat brain. The new model involved injection of viral vectors carrying human α-synuclein DNA into the vagus nerve in the rat neck, retrograde transport of the vectors, transduction of neurons and overexpression of α-synuclein within neuronal cell bodies and neurites in the medulla oblongata. Results provided important insight into mechanisms of α-synuclein accumulation, propagation and pathology. α-Synuclein overexpression was sufficient to trigger its interneuronal transfer into axonal projections innervating the medulla oblongata and connecting it to rostral sites in the pons, midbrain and forebrain. This effect was time- and concentration-dependent and was accompanied by evidence of protein fibrillation and neuritic pathology.

IMPACT:

Our findings provide direct evidence of long-distance α-synuclein propagation that could explain, at least in part, the caudo-rostral pattern of disease progression in PD. Data also demonstrate a relationship between increased α-synuclein expression, ascendant protein spreading and neuronal abnormalities, recapitulating mechanisms of likely relevance to PD pathogenesis. Future investigations using this new rat model will likely further our understanding of α-synuclein pathophysiology and contribute to the development of therapeutic strategies targeting α-synuclein accumulation and propagation.

### Histological quantifications

All evaluations were made by investigators blinded to treatment/experimental group. To determine the number of hα-syn-positive neurites, counts were made using sections at pre-defined Bregma coordinates (Paxinos & Watson, 2009): Bregma −9.60 mm in pons, −7.80 mm in caudal midbrain, −6.00 mm in rostral midbrain and −2.64 mm in forebrain.

The volume of hα-syn-immunoreactive swellings was measured in MO, pons, midbrain and forebrain at 8 and 18 weeks post vagal injection. For each region and time point, data were obtained and pooled from 5 animals. Tissue sections were visualized with a 63x oil immersion objective (Numerical aperture = 1.4) on either a Nikon Eclipse 90i (Nikon) or an Olympus IX2 UCB microscope equipped with Stereo Investigator software version 9 (MBF Biosciences). A guard zone thickness of 1 µm was set at the top and bottom of each section. For MO, measurements were carried out on a section at the level of the obex. After delineating the dorso-medial region of the left hemisphere, sampling parameters were set to count 4% of the area. For the pons (section taken at Bregma −9.60 mm), 68% of the entire left hemisphere was sampled. For the midbrain (Bregma −6.0 mm) and forebrain (Bregma −2.64 mm), 100% of the left hemisphere was analyzed. The stereological isotropic nucleator probe (Stereo Investigator) was used as previously described (Gundersen et al, 1988). To estimate volumes, the nucleator probe was set to generate 4 random isotropic linear rays emerging from a user-defined center point within each varicosity. The points at which the 4 rays touched the perimeter of the swelling were also manually defined. Varicosities with a volume greater than 0.5 µm^3^ were included in data analysis.

The total number of tyrosine hydroxylase-positive neurons was estimated using an unbiased stereological quantification method and employing the optical fractionator principle (Stereo Investigator) (Ulusoy et al, 2010). The SN was delineated at low magnification in every sixth section throughout the entire region. Counting was performed using a 63× Plan-Apo oil objective on an Olympus IX2 UCB microscope equipped with a MBF Mac6000 System stage (Microbrightfield) and a high precision encoder. The coefficient of error was calculated according to Gundersen and Jensen (1987), and values were <0.10.

### Reverse Transcription PCR (RT-PCR)

Samples for RT-PCR and quantitative RT-PCR (qRT-PCR) analyses were obtained from 40-μm fixed tissue sections. The dorso-medial quadrant of the left MO containing the DMnX was dissected and pooled from caudo-rostral, equally spaced (2 sections every 160 μm) sections at Bregma −14.76 to −12.48 mm. These samples were used to generate data in Fig 1. MO data in Fig 1 were obtained using DMnX-contained samples dissected from equally spaced (3 sections every 120 μm) sections at caudal (Bregma −14.76 to −14.00 mm), middle (Bregma −14.00 mm to −13.36 mm) or rostral (Bregma −13.36 mm to −12.48 mm) levels. In Fig 1, samples from the dorso-medial, dorso-lateral, ventro-medial and ventro-lateral pons were dissected from equally spaced (2 sections every 160 μm) sections between Bregma −10.32 and −9.00 mm. Control tissue and reference samples were generated using the same dissection procedures, and control brains were obtained from sham-operated rats.

Total RNA was extracted using the “RecoverAll Total Nucleic Acid Kit” (Ambion), according to manufacturer's instructions. RNA yield was measured by absorbance reading. cDNA was synthesized using 150 ng of total RNA (SuperScript VILO Master Mix, Invitrogen). The following primer sequences were used: total (human plus rat) α-syn: 5′ tggttttgtcaaaaaggaccag forward and 5′ ccttcctcagaaggcatttc reverse; rat-only α-syn: 5′ gagttctgcggaagcctagagagc forward (aligning on the 5′ UTR region of rat α-syn mRNA) and 5′ gttttctcagcagcagccacaactcc reverse; WPRE: 5′ ccgttgtcaggcaacgtg forward and 5′ agctgacaggtggtggcaat reverse; hypoxantine phosphoribosyltransferase 1 (housekeeping gene): 5'gaccggttctgtcatgtcg forward and 5'acctggttcatcatcactaatcac reverse.

In some instances (Fig 1), RT-PCR products were run on a 1.5% agarose gel. For qRT-PCR (Fig 1), analyses were performed in triplicates, using 1 μl of cDNA and Power SYBR Green Master Mix (Applied Biosystems). Relative quantities (fold changes) were obtained after normalization to the expression of the housekeeping gene and calibration to the corresponding reference sample.

### Statistical analyses

Statistical analyses were carried out with JMP Pro Statistical software (version 10.0.0; SAS Institute). For normally distributed data, means between two groups were compared with two-tailed *t*-test and comparisons of means from multiple groups were analyzed with one-way ANOVA followed by Tukey *post hoc* test or *t* test with Bonferroni correction. Non-normally distributed data were analyzed with non-parametric Wilcoxon rank sum test. Statistical significance was set at *P* < 0.05.

## Supplementary material

As a service to our authors and readers, this journal provides supporting information supplied by the authors. Such materials are peer reviewed and may be re-organized for online delivery, but are not copy-edited or typeset. Technical support issues arising from supporting information (other than missing files) should be addressed to the authors.
