# Supplementary material for: Caudo-rostral brain spreading of α-synuclein through vagal connections
Source: EMBO Mol Med. 2013 May 23;5(7):1051–9. doi: 10.1002/emmm.201302475 (PMC3721477; doi:10.1002/emmm.201302475)
Supplement: Supplementary file 1 [file emmm0005-1051-SD1.pdf]

EMBO Molecular Medicine

Caudo-rostral brain spreading of α-synuclein through vagal connections

Ayse Ulusoy, Raffaella Rusconi, Blanca I Pérez-Revuelta, Ruth E Musgrove, Michael Helwig, Bettina Winzen-Reichert, Donato A Di Monte

Corresponding author: Donato Di Monte, German Centre for Neurodegenerative Disease (DZNE), Bonn, Germany

Review timeline:

Submission date:	09 January 2013
Editorial Decision:	06 February 2013
Revision received:	05 March 2013
Editorial Decision:	03 April 2013
Revision received:	08 April 2013

Transaction Report:

(Note: With the exception of the correction of typographical or spelling errors that could be a source of ambiguity, letters and reports are not edited. The original formatting of letters and referee reports may not be reflected in this compilation.)

Editors: Natascha Bushati, Céline Carret

1st Editorial Decision 06 February 2013

Thank you for the submission of your manuscript to EMBO Molecular Medicine. We have now heard back from the three referees whom we asked to evaluate your manuscript. Although the referees find the study to be of potential interest, they also raise a number of concerns specifically regarding the lack of important controls.

As you will see from the comments below, while Referees 2 and 3 are overall supportive, they nevertheless ask for text clarifications, tune down of the main claims and importantly, together with Referee 1, require to perform additional controls regarding the spread of alpha-synuclein vs. the spread of the virus. We feel that this is a critical point and would particularly insist on this issue. In addition, Referee 1 suggests a number of experiments that would strenghen the data.

Given these evaluations, we would be happy to consider a revised version of your manuscript, with the understanding that the referees' concerns must be fully addressed and that acceptance of the manuscript may entail a second round of review.

Please note that it is EMBO Molecular Medicine policy to allow a single round of revision only and that, therefore, acceptance or rejection of the manuscript will depend on the completeness of your responses included in the next, final version of the manuscript.

As you know, EMBO Molecular Medicine has a "scooping protection" policy, whereby similar findings that are published by others during review or revision are not a criterion for rejection. However, I do ask you to get in touch with us after three months if you have not completed your revision, to update us on the status. Please also contact us as soon as possible if similar work is published elsewhere.

I look forward to receiving your revised manuscript

***** Reviewer's comments *****

Referee #1 (Comments on Novelty/Model System):

In this study, the authors inject an adeno-associated viral vector in the left vagus nerve in order to express GFP or human alpha-synuclein and observe the pattern of human alpha-synuclein progression. While supporting previous pathological and experimental findings regarding PD pathophysiology, this animal model does not provide additional information on PD's aetiology.
It is not the first atudy that shows alpha-synuclein trancellular transport. There are at least four groups showing this in vivo and in vitro. Two of them are mentioned in the paper, the other two (Freundt et al. 2012) (Pan-Montojo et al. 2012) are not and should be discussed.
Moreover, the authors seem to forget some literature showing PD pathology (alpha-synuclein accumulation) in the enteric nervous system from biopsies of PD patients and postmortem tissue (Lebovier et al. 2010, Wakabayashi et al. 1997) and in the intermediolateral column of the spinal cord (Braak et al. 2007).
In order to further validate their model, they should analyze the presence of human alpha-synuclein in the ENS. A recent study has shown that alpha-synuclein could be tranported anterogradelly and release to the extracellular space (Freundt et al. 2012). Therefore, it would be interesting to analyze the presence of human-alpha-synuclein in the enteric nervous system of their animals. If found, this could provide one explanation for the above-mentioned alterations observed in patients.
If not, it would be necessary to inject the human-alpha-synuclein containing adenovirus in different regions of the enteric nervous system to analyze its effect and test whether there is progression of alpha-synuclein to synaptically connected structures (i.e. the DMnX and the spinal cord) as seen in PD patients.

Further, it is known that viral particles and RNA can be retrogadelly transported and transferred between cells. In order to analyze the presence of human-alpha-synuclein RNA in other brain regions, the authors performed RT-PCR of different pons regions. However, they missed an important control, as they did not, at least it is not stated anywhere, analyze the presence of human-alpha-synuclein RNA in the MO contralateral to the injection (right side). They do state that human alpha-synuclein staining can be observed on both sides and they suggest that this is due to "some of these fibers crossed the midline and innervated the contralateral (uninjected) MO". Therefore, either they state that GFP does not follow this pattern or it would be necessary to determine whether human-alpha-synuclein RNA is to be observed on this side.

Finally, they did not find human-alpha-synuclein in the substantia nigra, any PD-related alterations (i.e. alpha-synuclein accumulation) or a reduction in the number of dopaminergic neurons. They should at least look into these aspects and comment it in the discussion.

All these comments are explained in more detail in the remarks to be sent to the authors.

Referee #1 (General Remarks):

In this study, the authors inject an adeno-associated viral vector in the left vagus nerve in order to express GFP or human alpha-synuclein. They then follow the appearance of human-alpha-synuclein in different regions of the CNS at two different time points (8 and 18 weeks). Interestingly, their results show that human alpha-synuclein progresses from the vagus nerve and the DMnX to nervous structures localized in mesencephalic structures and the forebrain (e.g. the locus coeruleus or the amygdala) in a topographic predictable manner similar to that described by Braak and colleagues in

PD patients.

General comments:

The results of the submitted manuscript confirm to a certain extent studies published by other groups. There are at least four studies showing complementary results in in vivo and in vitro models. Two of them are mentioned in the paper and have shown the interneuronal spreading of alpha-synuclein in the brain and in vitro (Desplats et al. 2009 and Luk et al. 2012). The third and fourth are more recent and are not mentioned in the paper. The third study shows the retrograde and anterograde transport of alpha-synuclein in cortical neurons cultured in microfluidic chambers (Freundt et al. 2012). The fourth shows that the resection of the sympathetic and parasympathetic nerves stop the progression of the pathology in an oral rotenone mouse model and demonstrate the intercellular transport of alpha-synuclein between enteric and sympathetic neurons using a co-culture based on the Campenot chamber (Pan-Montojo et al. 2012).

The present study, while providing new evidence that supports previous pathological (from PD patients) and experimental studies regarding PD's pathophysiology and progression, does not fully reproduce the lesions seen in PD patients or provide additional information on PD's aetiology. Indeed useful for studies regarding alpha-synuclein transmission, overexpression of human-alpha-synuclein is a very artificial state that mimics only two of the possible genetic alteration found in PD patients (duplications and triplications of the PARK-1 (alpha-synuclein) gene (Singleton et al. 2003, Farrer et al. 2004)). Thus, representing less than 1% of PD cases.

Moreover, the authors did not analyze the presence of PD-related alterations in the peripheral nervous system or the substantia nigra (at least they do not mention it). Different studies have shown the importance and frequency of the lesions observed in the enteric nervous system of PD patients. Lewy bodies and general alpha-synuclein accumulations have been detected in both biopsies and postmortem tissue in this region (Lebovier et al. 2010, Wakabayashi et al. 1997). Further studies have also observed these lesions in the intermediolateral column of the spinal cord (Braak et al. 2007).

In this direction, it would be interesting to analyze the presence of human alpha-synuclein in the ENS. A recent paper showed that neurons in the DMnX tend to have increased oxidative stress because of their pacemaker activity and an increased calcium flux (Goldberg et al. 2012). Furthermore and as mentioned above, a recent study has shown that alpha-synuclein could be tranported anterogradelly and release to the extracellular space (Freundt et al. 2012). Therefore, the authors should analyze the presence of human-alpha-synuclein in the enteric nervous system of animals injected with human-alpha-synuclein. If found in the ENS, it could provide an explanation for the alterations observed in patients. If not, it would be important to determine whether the injection of the human-alpha-synuclein containing adenovirus in different regions of the enteric nervous system could mimic the progression of alpha-synuclein observed in PD patients (i.e. progression to the DMnX and the spinal cord and upwards).

Finally, the authors did not find human-alpha-synuclein or any PD-related alterations in the substantia nigra (i.e. alpha-synuclein accumulation, motor alterations or a reduction in the number of dopaminergic neurons). Did the authors look into this? They should at least investigate these aspects and, even if the results are negative, comment it in the discussion.

Specific comments:

Results

1) "In agreement with earlier reports.... some of these fibers crossed the midline and innervated the contralateral (uninjected) MO". What is innervating what? Do the authors mean that the

contralateral DMnX gives some fibers to the transfected vagal nerve or that the ipsilateral DMxV innervates the contralateral MO? MO refers to the medulla oblongata in general, please specify region within MO.
2) An important control is missing or was not mentioned in the text. It is known that RNA and viral particles can be transported between cells. In order to verify that the presence of human-alpha-synuclein in the contralateral MO is not due to the presence of human-alpha-synuclein RNA, the contralateral MO should be microdissected (if possible avoding the nerves that belong to the contralateral vagal nerve) and RT-PCR should be performed. If I understood correctly from the Material and Methods, the whole MO was used for PCR. One other way of testing this is by analyzing the presence of GFP in these fibers and the contralateral MO regions. Did the authors look into this? They mention that GFP is confined to the MO but they do not specify the pattern.
3) Some studies suggest that transcellular-transported alpha-synuclein can act as a nucleating agent (Desplats et al. 2009). It would be interesting to stain against rat alpha-synuclein to see whether this colocalization can also be observed in this animal model.
4) Figure 1B Please specify which photo corresponds to DMnX and which one to the solitary tract.
5) Figure 1F. What is compared? Almost all region of the MO seem to be expressing significant higher amounts than those in the pons.
6) Supplementary Figure 1 could be included in Figure 1.
7) Supplementary Figures 2 to 4: Please provide lower magnification images.

Referee #2 (General Remarks):

This manuscript presents provocative data on the ability to experimentally reproduce some aspects of the spread of alpha-synuclein pathology thought to occur in early stages of Parkinson's disease (PD). These are important data because post-mortem observations in human are by nature correlative and require experimental validation to support mechanistic hypotheses.

Overall the study is well executed but some additional controls would strengthen the manuscript. In addition, a more balanced description of the results is warranted.

1. The main point of the study is to demonstrate that alpha-synuclein can spread from nuclein in the ventral medulla to rostral brain regions that are affected in PD. Unfortunately, the complete pattern of pathology observed in PD was not reproduced, notably the spread to neurons of the substantia nigra which harbor alpha-synuclein pathology and die in PD. The absence of nigral pathology and cell death is per se NOT a reason to discount this study or the value of this model. Indeed, although central to PD pathology, nigral pathology and cell death are only one element of the disorder. However, the authors need to acknowledge this limitation openly and tone down their conclusions accordingly.

2. The authors are correct in noting that their obseverations of a predominantly axonal pathology reproduced observations in post-mortem brains; however, alpha-synuclein aggregates are also present in the cytoplasm of neuronal cell bodies in PD, and does not seem to have been observed in this model. Again, this limitation should be acknowledged and discussed.

3. The authors interpret their data as due to the spread of alpha-synuclein form neurons to neurons; yet they express alpha-synuclein by means of a virus. Their claim that the results are not due to viral spread, and are specific for alpha-synuclein, is primarily based on the absence of expression of GFP, used as a control, in regions that show immunostaining for alpha-synuclein. GFP levels can be low, and difficult to detect even by immunohistochemistry. In addition, GFP is much larger that alpha-synuclein. Furthermore, small differences in level of expression (1.6 versus 1.9) seem to have dramatic effects on the level of transfer of alpha-synuclein; accordingly, GFP may not have been expressed at high enough level to show the spread observed for alpha-synuclein, be too big for tranfer or be expressed in rostral regions at levels too low for detection. Is it possible to strengthen the conclusions by detecting other viral antigens or by expressing a protein of similar size as alpha-

synuclein that can be detected with good antibodies, and can be expressed at high levels? If this is experimentally not feasible, this point remains important to discuss.

4. The conclusion implies that the data reveal a novel concept in the pathology of PD; this is not the case as the notion described at the end of the paper has been proposed by numerous investigators over the last 10 years based on genetic, pathological and experimental evidence. The current data add interesting support to this hypothesis and provide novel experimental conditions to further probe mechanisms but they are not per se novel.

Referee #3 (General Remarks):

Here Ulusoy et al. develop a rat model where human a-syn is over-expressed only in medullary neurons, and ask if rostral spreading of a-syn occurs in such a setting. The model is relevant to human pathology as numerous neuropathologic studies suggest a similar caudo-rostral spreading in humans. The paper is clearly written and the quality of the data is good. The following points need to be addressed:

1. Fig. 2E makes the most critical point of the paper, and the authors need to show the primary data to substantiate their points. Though the spreading of a-syn pathology is clear from the quantification (above) and the mapping of a-syn +ve areas (as red dots), it would be nice to see a whole section from each zone to go along with these data. This would be similar to what is shown in fig. 1A, but for Pons, cMB, rMB and FB (perhaps with inset/zoom images).

2. The authors mention in passing that high-expressers show only scant spreading, but the data is not shown. There is also no real discussion of this issue, other than the mention that the spreading is "...concentration dependent". It is possible that higher expression leads to more aggregation of a-syn in proximal neurons, essentially sequestering the protein in caudal compartments and limiting the spread. The authors could ask if the axonal varicosities/aggregates they see in high-expressers are greater, which would provide some correlative support to this idea (though more experiments are obviously needed to fully explore this, for example use of the A53T/C-term del etc. mutants in the low-expresser setting where aggregation would presumably be faster - suggested as future experiments, not needed for this study). At least there should be a discussion as to why a higher expression may lead to lesser spread.

3. Throughout the paper, the authors use the term "diffusion" to describe the spreading. However physical laws dictate that in an axon, diffusion exponentially decays over time and thus in long axons, proteins from the soma will never reach the tip of the axon by diffusion alone (let alone spread from one neuron to another - hence the need for axonal transport). Similar things have also been experimentally demonstrated in old papers (for example see Koike and Matsumoto, Neuroscience Research, 2 (1985) 281-285). Though this term has been used by others in the literature to explain the spread, in my opinion this is an incorrect usage of the term.

1st Revision - authors' response 05 March 2013

March 5, 2013

Dear Dr. Carret:

Thank you for your consideration of this revised version of our manuscript entitled "Caudo-rostral brain spreading of α-synuclein through vagal connections" (EMM-2013-02475). In your message summarizing the comments of the Reviewers, you emphasized the importance of performing additional control experiments to rule out spreading of the virus. As further detailed below, we have now included data obtained using tissue from the side of the brain contralateral to viral injections and analyzing samples for the presence of additional viral markers. Data confirm and further support our original conclusion, i.e. that overexpression of human α-synuclein triggers neuron-to-neuron passage and long-distance spreading of the protein.

We also thank the Reviewers for providing valuable comments, which have been addressed as follows:

<u>Reviewer 1:</u>

Specific comments:

1a. *"What is innervating what? Do the authors mean that the contralateral DMnX gives some fibers to the transfected vagal nerve or that the ipsilateral DMxV innervates the contralateral MO? MO refers to the medulla oblongata in general, please specify region within MO."*

In response to the Reviewer's specific comment #1, we modified the paragraph on pages 4-5 of the manuscript describing the innervation of the medulla oblongata through the vagus nerve. We have also labeled more clearly areas of the medulla oblongata connected to the vagus nerve in panels A, C, D, E and F of Figure 1.

2a. *"In order to verify that the presence of human-alpha-synuclein in the contralateral MO is not due to the presence of human-alpha-synuclein RNA, the contralateral MO should be microdissected (if possible avoiding the nerves that belong to the contralateral vagal nerve) and RT-PCR should be performed."*

To address the important issue raised by this Reviewer in his/her second specific comment, we have analyzed the medulla oblongata from the side of the brain contralateral to viral injections for the presence of human α-synuclein mRNA. The new data are described at the end of page 5 and shown in panel G of Figure 1. They confirm that only human α-synuclein <u>protein</u> but not <u>mRNA</u> is detectable in these contralateral samples. The lack of virus-derived mRNA for human α-synuclein provides additional evidence of transport of the protein in the absence of viral particle spreading.

2b. *"They mention that GFP is confined to the MO but they do not specify the pattern."*

As suggested by the Reviewer, we clearly indicate in the text (end of 1st paragraph on page 5) that the pattern of distribution of efferent and afferent vagal fibers in the MO was virtually indistinguishable between animals injected with hα-syn- or GFP-carrying AAV vectors. We have also added a low magnification image showing this pattern in Supporting Information Fig 1.

3. *"It would be interesting to stain against rat alpha-synuclein."*

To determine whether exogenous α-synuclein is capable of "seeding" the endogenous protein, tissues should be stained with a highly specific antibody capable of labeling rat but not human α-synuclein. Such an antibody is not currently commercially available. We have also tested a variety of antibodies obtained from non-commercial sources and found them unable to distinguish between rat and human immunoreactivity.

4. *"Figure 1B Please specify which photo corresponds to DMnX and which one to the solitary tract."*

   As already mentioned, panels in Figure 1 have been more clearly labeled.

5. *"Figure 1F. What is compared? Almost all region of the MO seem to be expressing significant higher amounts than those in the pons."*

   The purpose of these analyses was indeed to show that levels of human α-synuclein are increased throughout the medulla oblongata but not in the pons. The data are consistent with (i) the distribution of vagal projections reaching the medulla oblongata but not the pons, and (ii) the lack of spreading of viral particle from the medulla oblongata to the pons (1st paragraph on page 6).

6. *"Supplementary Figure 1 could be included in Figure 1."*

   Supporting Information Fig 1 (GFP expression) shows images that mirror images in Fig 1 (human α-synuclein expression). We believe that, for this reason, combining the two figures could potentially lessen clarity of the illustration.

7. *"Supplementary Figures 2 to 4: Please provide lower magnification images."*

   Because the spreading of human α-synuclein to pons, midbrain and forebrain affects discrete axonal projections, low magnification images are not very illustrative. Nevertheless, we have added low magnification images in Figure 2 (panels F-I) to better define the anatomical sites of α-synuclein spreading.

General comments:
In his/her general comments, this Reviewer points out that a recent paper (Freundt et al.) concerning the *in vitro* transport of α-synuclein was not included in the original list of references. This has now been rectified. The Reviewer also refers to the hypothesis that α-synuclein pathology in Parkinson's disease may commence in the peripheral nervous system and reach the CNS *via* the vagus nerve. This hypothesis is now briefly discussed in the Introduction section of the revised manuscript (page 3) where we also refer to another published paper quoted by the Reviewer (Pan-Montojo et al.). Finally, Reviewer 1 raises the issue of spreading of α-synuclein toward the substantia nigra. As further described below, an entire new section discussing this topic has been added to the present manuscript (pages 8-9).

Reviewer 2:

The introductory comments of Reviewer #2 underscore the importance of our study reproducing a critical aspect of α-synuclein pathophysiology in early stages of Parkinson's disease. This Reviewer's suggestions have been addressed as follows:

1. *"The main point of the study is to demonstrate that alpha-synuclein can spread from nuclei in the ventral medulla to rostral brain regions that are affected in PD. Unfortunately, the complete pattern of pathology observed in PD was not reproduced, notably the spread to neurons of the substantia nigra which harbor alpha-synuclein pathology and die in PD. The absence of nigral pathology and cell death is per se NOT a reason to discount this study or the value of this model. Indeed, although central to PD pathology, nigral pathology and cell death are only one element of the disorder. However, the authors need to acknowledge this limitation openly and tone down their conclusions accordingly."*

   The spreading of α-synuclein described in our manuscript does not reach the substantia nigra pars compacta. This could be a limitation of this animal model. It could also reflect the fact that a longer (>18 weeks) interval of time may be required for the exogenous protein to reach areas of the brain (including the substantia nigra) that do not have direct anatomical connections with the medulla oblongata. These different possibilities are discussed in the revised paper (pages 8-9).

2. *"The authors are correct in noting that their observations of a predominantly axonal pathology reproduced observations in post-mortem brains; however, alpha-synuclein aggregates are also present in the cytoplasm of neuronal cell bodies in PD, and does not seem to have been observed in this model. Again, this limitation should be acknowledged and discussed."*

   Prompted by this Reviewer's comments, we have also added a discussion on the lack of overt accumulation of exogenous α-synuclein within neuronal cell bodies (pages 8-9).

3. *"Is it possible to strengthen the conclusions by detecting other viral antigens or by expressing a protein of similar size as alpha-synuclein that can be detected with good antibodies, and can be expressed at high levels? If this is experimentally not feasible, this point remains important to discuss."*

   To strengthen our conclusion that the spreading of human α-synuclein is unlikely to be due to propagation of the viral particles, we designed primers capable of binding a specific component of our viral vector, i.e. the woodchuck hepatitis virus post-transcriptional regulatory element (WPRE). Using these primers to amplify this viral marker, we were able to show that mRNA for WPRE was present (as expected) in the medulla oblongata but absent in the pons. These new data are now described on page 6 (1st paragraph) and shown in panel J of Figure 1.

4. *"The conclusion implies that the data reveal a novel concept in the pathology of PD; this is not the case as the notion described at the end of the paper has been proposed by numerous investigators over the last 10 years based on genetic, pathological and experimental evidence. The current data add interesting support to this hypothesis and provide novel experimental conditions to further probe mechanisms but they are not per se novel."*

   Comments on the novelty of our findings have been toned down.

Reviewer 3:

This Reviewer also emphasizes the relevance of the animal model described in this study to human pathology. This Reviewer's suggestions have been addressed as follows:

1. *"Fig. 2E makes the most critical point of the paper, and the authors need to show the primary data to substantiate their points. Though the spreading of a-syn pathology is clear from the quantification (above) and the mapping of a-syn +ve areas (as red dots), it would be nice to see a whole section from each zone to go along with these data."*

   The Reviewer suggests adding whole sections from different rat brain regions (pons, midbrain and forebrain) to better define the sites of α-synuclein spreading. We did so in panels F-I of the new Figure 2. These panels also illustrate that the spreading of the exogenous protein affects sparse axonal projections that can only be clearly appreciated at higher magnifications (see comments above).

2. *"The authors mention in passing that high-expressers show only scant spreading, but the data is not shown. .... At least there should be a discussion as to why a higher expression may lead to lesser spread."*

   Statements in the previous manuscript gave the erroneous impression that spreading was less pronounced in higher as compared to lower expressor rats. The opposite is of course true (see, for example, Supporting Information Fig 4). We thoroughly reviewed the new manuscript to ensure clarity.

3. *"Throughout the paper, the authors use the term "diffusion" to describe the spreading. However physical laws dictate that in an axon, diffusion exponentially decays over time and thus in long axons, proteins from the soma will never reach the tip of the axon by diffusion alone (let alone spread from one neuron to another - hence the need for axonal transport). ... Though this term has been used by others in the literature to explain the spread, in my opinion this is an incorrect usage of the term."*

   We agree with this Reviewer who pointed out that the term "diffusion" (to indicate spreading) may not be correct and cause some confusion. This term has been substituted throughout the revised manuscript.

We believe that we have effectively addressed the Reviewers' suggestions/comments in this new version of the manuscript and that the described changes have strengthened the data and conclusions of this important study. Please do not hesitate to contact me should any additional information be required.

Best regards,

Donato A. Di Monte, M.D.

2nd Editorial Decision 03 April 2013

Thank you for the submission of your manuscript to EMBO Molecular Medicine. We have now heard back from the three referees whom we asked to evaluate your manuscript. Although the referees find the study to be of potential interest, and two referees are now happy with the manuscript, referee #1 still raises a number of concerns already raised before and not addressed in the revision.

We would like to give you a last chance to address these issues. Please pay particular attention to the comments made by referee #1 in this review but also under general comments, in his/her previous report. We feel that some of these issues are very relevant to PD clinical symptoms and pathology, therefore they should be convincingly addressed in the next final version of the manuscript.

I look forward to seeing a revised form of your manuscript as soon as possible.

***** Reviewer's comments *****

Referee #1 (General Remarks):

Despite the fact that the authors have not even mentioned in their answers to the reviewers some of the questions raised, the lack of human-alpha-synuclein RNA in the MO contralateral to the transfected vagal nerve does make their result more consistent.

However, the author have addressed only few of my and others previous comments. The comments regarding the presence of human alpha-synuclein in the enteric nervous system was not addressed at all. Also, as different reviewers have asked, the comments regarding the results in the substantia nigra (cell count) or any functional tests are still missing and were not addressed or mentioned in the answers to the reviewers. The fact that the authors do not see any human alpha-synuclein in the substantia nigra does not necessarily mean that the substantia nigra is not affected. If the authors believe that, at a later time point, they will observe alpha-synuclein pathology in the substantia nigra, maybe they should wait until this occurs to re-submit the manuscript. The appearance of alterations (at least the ones investigated) in the substantia nigra is relevant to PD clinical symptoms and pathophysiology. Any PD's animal model should affect the substantia nigra in one way or another.

Finally,in case that the manuscript is finally accepted, the authors should further discuss their results in relation to the ENS spreading hypothesis. The observation that human-alpha-synuclein also affects the contralateral side should also be better discussed, would this also be the case if the pathology should start in the vagal terminals innervating the ENS or other organs? If I understood correctly, the contralateral spreading is due to the transfection of sensory vagal nerves. What nerves exactly? the visceral sensory (afferent general visceral), the visceral sensory (afferent special visceral) or the somatic sensory nerves? If, for example, somatic sensory, how is it relevant to PD if they normally innervate the skin?

This study is now correct from the technical point of view. It also gives some insights in the way the pathology progresses and its relation to Braak's staging of the disease. However, as some important points and experiments are missing, I cannot be sure of the impact that this work will have on the scientific community. Therefore, I cannot give a clear recommendation to publish this work, this should be evaluated by the editor.

Referee #2 (General Remarks):

The authors have thoughtfully and convincingly addressed the previous concerns.

Referee #3 (General Remarks):

My critiques have been addressed.

2nd Revision - authors' response 08 April 2013

Thank you very much for your letter and for the opportunity to further revise our manuscript. We are very pleased that Referees #2 and #3 found that the first revision of the paper "*thoughtfully and convincingly addressed the previous concerns*". Reviewer #1 also acknowledged *"the study is now correct from the technical point of view. It also gives insights in the way the pathology progresses and its relation to Braak's staging of the disease"*. Despite these positive general comments, Reviewer #1 was not completely satisfied with the improvements made to the original submission. We carefully assessed this Referee's remarks and addressed his/her specific request for additional data concerning the integrity of nigral neurons. Other comments of this Reviewer relate to the discussion of our findings and their potential impact. We provide a detailed explanation/justification of our viewpoint in the paragraphs below and, when necessary, have made changes in the text of the revised manuscript to further clarify data evaluation.

1. "*The results in the substantia nigra (cell count) or any functional tests are still missing. The fact that the authors do not see any human alpha-synuclein in the substantia nigra does not necessarily mean that the substantia nigra is not affected*"

We have now addressed this issue by performing stereological cell counting of nigral dopaminergic cells. Results of these measurements showing lack of cell loss are reported on page 8 of the revised manuscript.

The lack of nigral pathology is interpreted by this Reviewer as a finding that lowers the relevance of our study because *"any PD animal model should affect the substantia nigra"*. We respectfully disagree with this opinion. We do not claim that our model reproduces full-blown Parkinson's disease. Instead, our work demonstrates (for the first time in an animal model) long-distance spreading of alpha-synuclein from the medulla oblongata to more rostral brain regions following anatomically interconnected pathways. This is likely to reflect early stages of disease development that, even in humans, do not involve the substantia nigra. We strongly believe that, by recapitulating early pathogenetic events, the paradigm described in our manuscript is unique among other currently available models and will be instrumental for further investigations into mechanisms of alpha-synuclein pathology and strategies to counteract it. Please note that the other Referees concur with this assessment and have praised the value of our new model that *"reproduces some aspects of the spread of alpha-synuclein pathology thought to occur in early stages of Parkinson's disease"*.

2. *"The observation that human alpha-synuclein also affects the contralateral side should be better discussed"*

The discussion concerning the presence of human alpha-synuclein in the medulla oblongata contralateral to viral injection has now been expanded and additional references are given (end of page 4 and beginning of page 5). In particular, we emphasize the fact that the pattern of transgene expression strictly follows the predicted anatomical distribution of efferent and afferent fibres forming the vagus nerve. It involved (i) the dorsal motor nucleus of the vagus nerve and the nucleus ambiguus only on the side of the brain ipsilateral to viral injection, and (ii) afferent fibres originating from the inferior vagal ganglion and reaching the nucleus of the tractus solitarius bilaterally. Earlier anatomical studies have mapped vagal connections to the medulla oblongata after unilateral injection of tracers into the rat vagus nerve. The remarkable similarity between the pattern of transgene distribution in our investigation and the pattern of fiber and perikarya labeling in these previous reports provides strong basis for our interpretation of findings described on page 4 and 5 of the revised manuscript.

The Reviewer suggests a potential alternative explanation concerning the presence of human alpha-synuclein on the side of the brain contralateral to AAV injection: *"Would this also be the case if the*

pathology should start in the vagal terminals innervating the ENS and other organs?" Several lines of evidence are at odds with such explanation. First, as detailed above, the pattern of transgene expression is highly consistent with transduction of fibres and perikarya after unilateral injection of the virus. Second, spreading of alpha-synuclein from the enteric nervous system (ENS) would be expected to affect neuronal cells of the dorsal motor nucleus of the vagus nerve in the contralateral medulla oblongata. This, however, is not what we observed (page 4 and Figure 1). Finally, results of experiments in which rats were injected with AAV carrying GFP or alpha-synuclein showed a similar pattern of transgene distribution in the ipsilateral and contralateral medulla oblongata (page 5 and Supporting Information Fig 1). To conclude that the presence of exogenous protein in the contralateral medulla oblongata is a result of spreading from the ENS (and other organs) to the CNS, one would have to assume that not only human alpha-synuclein but also GFP is capable of interneuronal transmission and long-distance propagation. This assumption is obviously unfounded.

3. *"Authors should further discuss their results in relation to the ENS spreading hypothesis"*

We agree with this Reviewer that the possibility that pathological forms of alpha-synuclein may spread from the enteric nervous system (ENS) represents an interesting hypothesis (see page 3 of the manuscript). It is important to emphasize, however, that the present study was not designed to test this hypothesis, nor to develop a model mimicking ENS-to-CNS propagation of alpha-synuclein. In fact, we believe that the experimental paradigm used for our study may not necessarily be suitable for investigations into spreading from the ENS. In his/her comments to the original submission, this Reviewer suggested *"to determine whether the injection of the human alpha-synuclein containing adenovirus in different regions of the ENS could mimic the progression of alpha-synuclein observed in PD patients"*. Unfortunately, this approach would likely generate ambiguous results due to unrestrained diffusion of viral particles and relatively random cell transduction.

4. *"I cannot be sure of the impact that this work will have on the scientific community"*

At least two considerations may explain the different opinion concerning the relevance of this study that this Reviewer expresses as compared to our view and the view of the other two Referees. One of these considerations has already been discussed above. In point 1, we noted how our findings reproduce pathophysiological changes of alpha-synuclein relevant to early stages of Parkinson's disease development. Perhaps this aspect was not stressed enough in the original manuscript, lessening the enthusiasm of Reviewer #1 for our new animal model. Changes were made in the revised manuscript to underscore this point (see, for example, page 9). The second important consideration relates to the fact that, in our study, interneuronal transmission and long-distance propagation of alpha-synuclein were triggered by neuronal overexpression of the protein. This is a critical mechanistic observation. In his/her earlier comments to the original submission, Reviewer #1 appears to downplay the relevance of this finding by stating, for example, *"overexpression of alpha-synuclein is a very artificial state that mimics only two of the possible genetic alterations found in PD patients"*. We are not of the same opinion. A life-long increase in alpha-synuclein expression is indeed causally associated with rare genetic forms of parkinsonism. It is also true, however, that enhanced levels of alpha-synuclein underlie other conditions directly relevant to Parkinson's disease, such as (i) genetic variability in the promoter region of the alpha-synuclein gene (see, e.g., Maraganore et al. JAMA 296:661-670, 2006), (ii) aging (e.g., Li et al. J Neurosci 24:7400-7409, 2004), and (iii) exposure to neurotoxic agents (e.g., McCormack et al. J Neuropathol Exp Neurol 67:793-802, 2008). Thus, alpha-synuclein elevations, even if transient, could play an important role in the development and progression of sporadic Parkinson's disease and represent a feature shared by a variety of Parkinson's disease risk factors. This concept was already mentioned in the original version of the manuscript and has now been expanded on page 3 of the revised paper. As a corollary to this concept, our present observation of a direct relationship between alpha-synuclein elevation and protein propagation is likely to bear profound and widespread pathogenetic implications.

In conclusion, we would like to thank you again for giving us the opportunity to further improve our paper with the results of additional experiments and text modifications. We truly believe that all of the Reviewers' experimental concerns have been fully addressed and that other issues have been appropriately clarified. We hope that you will concur with our view and the view of two Reviewers

that the work described in this manuscript has significant relevance for human synucleinopathies and is therefore suitable for publication in your high-impact journal. Please do not hesitate to contact me should any additional information be required.
