# Supplementary material for: Caudo-rostral brain spreading of α-synuclein through vagal connections
Source: EMBO Mol Med. 2013 May 23;5(7):1051–9. doi: 10.1002/emmm.201302475 (PMC3721477; doi:10.1002/emmm.201302475)
Supplement: Supplementary file 2 [file emmm0005-1051-SD2.pdf]

Caudo-rostral brain spreading of α-synuclein through vagal connections

Ayse Ulusoy[1], Raffaella Rusconi[1], Blanca I Pérez-Revuelta[1], Ruth E Musgrove[1], Michael Helwig[1], Bettina Winzen-Reichert[1], Donato A Di Monte[1]*

[1]German Center for Neurodegenerative Diseases (DZNE), Bonn, Germany

*Correspondence to:

Prof. Dr. Donato A. Di Monte

Phone: +49 228 43302650

Fax: +49 228 43302689

E-mail: donato.dimonte@dzne.de

Table of contents
Supporting Information Figure Legends

Supporting Information Figure 1. Vagal injections of GFP-carrying AAV induce robust transduction of DMnX neurons

Supporting Information Figure 2. Hα-syn expression in the MO of a rat with suboptimal AAV transduction

Supporting Information Figure 3. Hα-syn spreads from the MO to catecholaminergic neurons in the pons

Supporting Information Figure 4. Hα-syn spreads from the MO to pons in rats with suboptimal AAV transduction

Supporting Information Figure Legends

Supporting Information Figure 1. Vagal injections of GFP-carrying AAV induce robust transduction of DMnX neurons. Representative MO sections from a rat killed at 2 weeks post viral injection were stained with an anti-GFP antibody. Caudo-rostral sections at corresponding Bregma levels show robustly stained neuronal bodies and neurites in the DMnX and nucleus ambiguus (Bregma -12.00 mm). Scale bar, 50 μm. A lower magnification section is shown at Bregma -13.76 mm.

Supporting Information Figure 2. Hα-syn expression in the MO of a rat with suboptimal AAV transduction. MO sections from a rat killed at 2 weeks after injection with hα-syn-carrying AAV were stained for hα-syn. Caudo-rostral sections at corresponding Bregma levels were visualized at lower (**A**) and higher (**B**) magnification. Higher-magnification images show neuronal bodies and neurites in the DMnX. The extent and intensity of immunoreactivity is evidently less pronounced in these images as compared to images in Figure 1 obtained from a high expressor animal.

Supporting Information Figure 3. Hα-syn spreads from the MO to catecholaminergic neurons in the pons. Tissue was collected from a high expressor rat killed 18 weeks after AAV injection. Confocal images show a pontine (coeruleus-subcoeruleus area) axon (arrows) co-labeled for tyrosine hydroxylase and hα-syn. The merged panel illustrates co-localization. Scale bar, 20 μm.

Supporting Information Figure 4. Hα-syn spreads from the MO to pons in rats with suboptimal AAV transduction. The number of neuritic projections immunostained with an anti-hα-syn antibody was counted in the pons of rats sacrificed at 18 weeks post vagal injection. Counts in low expressors (gray bars, n=6) are compared with values in high expressor animals (red bars, n=5). Counts are shown from the side of the brain ipsilateral and contralateral to viral injection. Data from high expressors are the same as those reported in Figure 2C,D. Mean ± SEM. **P<0.01, ***P<0.001 by two-tailed t test.

Supporting Information Figure 1

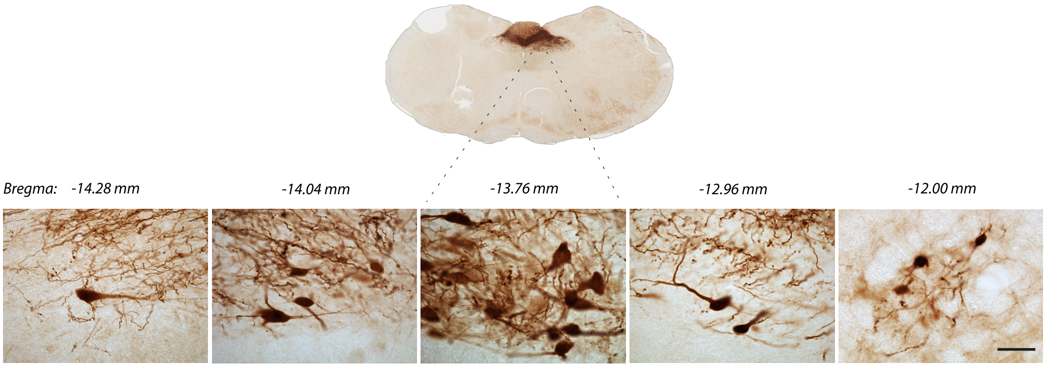

Supporting Information Figure 1. Vagal injections of GFP-carrying AAV induce robust transduction of DMnX neurons. Representative MO sections from a rat killed at 2 weeks post viral injection were stained with an anti-GFP antibody. Caudo-rostral sections at corresponding Bregma levels show robustly stained neuronal bodies and neurites in the DMnX and nucleus ambiguous (Bregma -12.00 mm). Scale bar, 50 µm. A lower magnification section is shown at Bregma -13.76 mm.

Supporting Information Figure 2

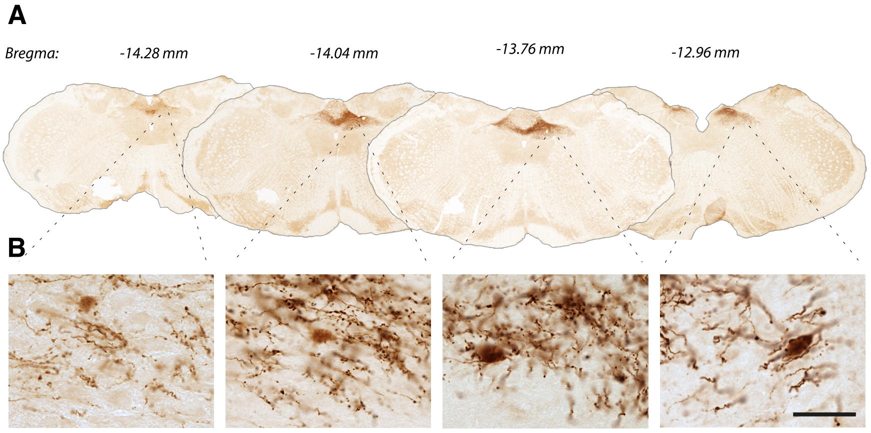

Supporting Information Figure 2. Hα-syn expression in the MO of a rat with suboptimal AAV transduction. MO sections from a rat killed at 2 weeks after injection with hα-syn-carrying AAV were stained for hα-syn. Caudo-rostral sections at corresponding Bregma levels were visualized at lower (A) and higher (B) magnification. Higher-magnification images show neuronal bodies and neurites in the DMnX. The extent and intensity of immunoreactivity is evidently less pronounced in these images as compared to images in Figure 1 obtained from a high expressor animal. Scale bar, 50 µm.

Supporting Information Figure 3

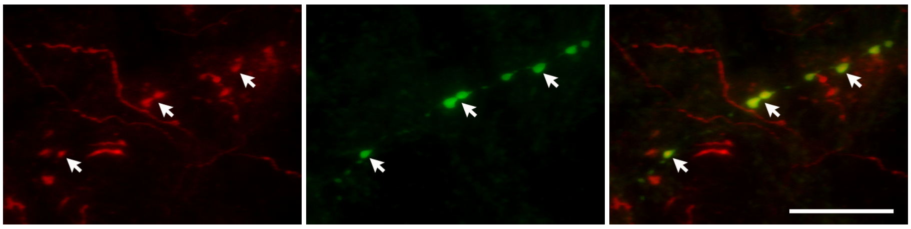

Supporting Information Figure 3. Hα-syn spreads from the MO to catecholaminergic neurons in the pons. Tissue was collected from a high expressor rat killed 18 weeks after AAV injection. Confocal images show a pontine (coeruleus-subcoeruleus area) axon (arrows) co-labeled for tyrosine hydroxylase and hα-syn. The merged panel illustrates co-localization. Scale bar, 20 μm.

Supporting Information Figure 4

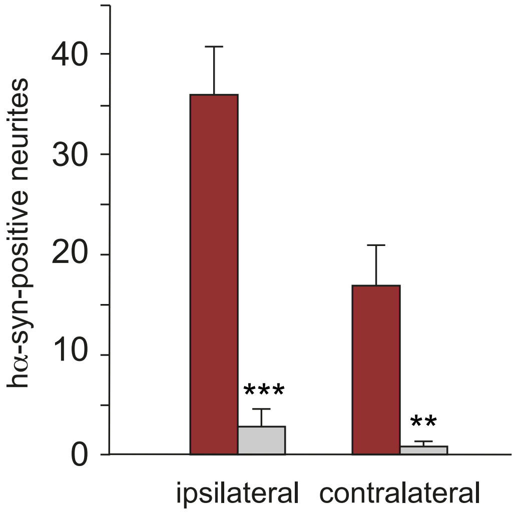

Supporting Information Figure 4. Hα-syn spreads from the MO to pons in rats with suboptimal AAV transduction. The number of neuritic projections immunostained with an anti-hα-syn antibody was counted in the pons of rats sacrificed at 18 weeks post vagal injection. Counts in low expressors (gray bars, n=6) are compared with values in high expressor animals (red bars, n=5). Counts are shown from the side of the brain ipsilateral and contralateral to viral injection. Data from high expressors are the same as those reported in Figure 2C,D. Mean ± SEM. **$P<0.01$, ***$P<0.001$ by two-tailed t test.
